# Envelope Dyadic Green’s Function for Uniaxial Metamaterials

**DOI:** 10.1038/s41598-019-55647-0

**Published:** 2019-12-27

**Authors:** Stanislav I. Maslovski, Hodjat Mariji

**Affiliations:** 1 Instituto de Telecomunicações e Departamento de Eletrónica, Telecomunicações e Informática, Universidade de Aveiro, Campus Universitário de Santiago, 3810-193 Aveiro, Portugal; 2 Instituto de Telecomunicações, DEEC FCTUC Pólo II - Pinhal de Marrocos, 3030-290 Coimbra, Portugal

**Keywords:** Metamaterials, Microwave photonics

## Abstract

We introduce the concept of the envelope dyadic Green’s function (EDGF) and present a formalism to study the propagation of electromagnetic fields with slowly varying amplitude (EMFSVA) in dispersive anisotropic media with two dyadic constitutive parameters: the dielectric permittivity and the magnetic permeability. We find the matrix elements of the EDGFs by applying the formalism for uniaxial anisotropic metamaterials. We present the relations for the velocity of the EMFSVA envelopes which agree with the known definition of the group velocity in dispersive media. We consider examples of propagation of the EMFSVA passing through active and passive media with the Lorentz and the Drude type dispersions, demonstrating beam focusing in hyperbolic media and superluminal propagation in media with inverted population. The results of this paper are applicable to the propagation of modulated electromagnetic fields and slowly varying amplitude fluctuations of such fields through frequency dispersive and dissipative (or active) anisotropic metamaterials. The developed approach can be also used for the analysis of metamaterial-based waveguides, filters, and delay lines.

## Introduction

Nowadays, there has been a growing interest in metamaterials (MMs) which are artificial composites used in various branches of science and engineering. After the pioneering works on the backward wave propagation in media^1–4^, many scientists have focused on the appealing features of MMs such as the negative refractive index of active and passive MMs^5–7^, the diffraction-unlimited imaging^8^, and the remarkable control over the electromagnetic fields^9^. These features allowed for fabrication of flat lenses^10^, invisible cloaking devices^11^, and perfect electromagnetic absorbers^12,13^. MMs have been employed for the modeling of general relativity effects with artificial black holes^14^ and to achieve frequency-agile or multi-band operation^15–17^, to mediate the repulsive Casimir force^18^, and to tune microwave propagation with light^19^. Another area in which MMs may have a broad impact that has recently attracted attention of science and technology is the near-field super-Planckian radiative heat transfer^20–22^, with applications for thermophotovoltaics^23,24^.

Note that the MMs typically operate in a narrow band around a fixed frequency. Therefore, when dealing with the amplitude and phase fluctuations of the electromagnetic fields in such MMs, it is appealing to construct a framework in which the generation and propagation of such excitations is described by linear operators acting directly on the slowly varying envelope of the excitations. In pursuing this goal, here we introduce the concept of the *envelope* dyadic Green’s function (EDGF) and develop a related formalism to deal with the electromagnetic fields with slowly varying amplitude (EMFSVA) in dispersive anisotropic MMs with known dyadics of permittivity ($\bar{\bar{\varepsilon }}$) and permeability ($\bar{\bar{\mu }}$). It has to be noted that, in this work, we only consider frequency dispersion and neglect any spatial dispersion effects.

Such formalism could be used, e.g. to study the dynamics of the electromagnetic fluctuations in MM-based radiative heat transfer (RHT) systems^22^, where the fluctuating electromagnetic field is created by the thermally agitated Johnson-Nyquist currents with spectral densities concentrated near the MM resonances. Besides the RHT problems, the EDGF approach is well suited for dealing with spatio-temporally modulated signals in dispersive uniaxial MMs, e.g. when studying the spatio-temporal transformations (refraction, deflection, focusing, etc.) of modulated Gaussian beams in flat lenses and hyperlenses based on such media.

Having these applications in mind, here we also derive the paraxial approximation of the EDGF method applicable to the uniaxial MM layers, in particular, MMs with extreme anisotropy and wire media^25^. Such MMs can guide the electromagnetic fluctuations along the optical axis over many wavelengths^26,27^, which makes the paraxial approximation a desirable feature of the EDGF method. Generalization to the case of bianisotropic media is as well possible. In the present article, we focus on the uniaxial anisotropic MMs, because they allow for a closed-form analysis. The advantage of the closed-form EDGF approach as compared to, e.g. traditional Green’s function methods or the full-wave numerical simulation methods, is in a great reduction of the computation time, because the analytical expressions for the EDGF matrix elements are derived as explicit functions of the space and time coordinates. The uniaxial MMs are also known for advantageous optical properties for sensing^28^, nonlinear optical applications^29^, and spontaneous emission control^30^. In particular, in extremely anisotropic uniaxial MMs the beam diffraction effects can be suppressed, which creates new opportunities for the flexible control of the RHT in MMs.

Note that the above-mentioned effects depend on the relation between the phase and group velocities in a MM. In passive media, the sign of the phase velocity, ${v}_{{\rm{ph}}}=\omega /k$, can be chosen with respect to the direction of the group velocity, ${v}_{{\rm{gr}}}=\partial \omega /\partial k$, which is typically selected to be always positive. This is rooted in the fact that, in low dispersive passive media, the directions of the energy and information transfer and the group velocity coincide. However, in active media, the signal (i.e., information) propagation direction does not necessarily coincide with the apparent energy flow direction, because such media can produce responses more energetic than the incoming signals. On the other hand, the information propagation direction in any media is fixed by causality, which means that the sign of the group velocity can be uniquely determined with respect to this direction. Under this convention — which we adopt in our work — the sign of the group velocity can vary depending on the material properties. Moreover, in dispersive media with loss, the group velocity can become complex. Although the real part of the complex group velocity can be still related to the envelope’s propagation speed, the interpretation of the imaginary part of the group velocity varies among different authors^31^.

In some situations, the unusual dispersive properties of the MMs result in superluminal or subluminal group velocities, in which case this quantity can be higher^32^ or extremely lower than the speed of light in a vacuum^33^. It can also become zero or negative^34–36^. These effects in MMs, as well as the negative refractive index, are well-accommodated within the framework of the causality principle^37–40^. In particular, the superluminal effects are due to interference between the nearby frequency components in a narrow-band signal that has experienced propagation through a medium with anomalous dispersion^41^. Such effects are essentially kinematic and are not associated with any superluminal exchange of information and (or) energy between a signal source and a receiver. As is known^37^, the transfer of information is associated with the propagation of the envelope’s precursor, rather than with the envelope’s maximum. The former always happens at a speed $v\le c$. In recent decades, superluminal and subluminal group velocities have attracted attention in nonlinear optics^42^, quantum communication^43^, photon controlling and storage^44–46^, precision sensing^47^, high-speed optical switching^48,49^, broadband electromagnetic devices and delay compensation circuits in ultra-high-speed communication systems^50^, and in high resolution spectrometers^51^. In this work, we examine the EDGF formalism on the hyperbolic MMs and media with gain, in order to demonstrate the applicability of the method to the wave processes in such media, including the negative refraction and focusing and the exotic superluminal processes in active media.

This article is organized as follows. We start from introducing the slowly varying complex amplitudes (SVA) of the electric and magnetic fields and writing the macroscopic Maxwell equations and the material relations in terms of these quantities. The EDGF is then found as the solution of the resulting 6-dyadic dynamic equation with a band-limited point source. Next, we obtain the EDGF matrix elements for the uniaxial anisotropic MMs and develop the paraxial approximation for the EDGF. In numerical examples, we apply the EDGF method to study characteristic effects associated with the propagation of spatio-temporally modulated signals in MMs (negative refraction) and in active media (superluminality), and confirm the applicability of the EDGF approach to these problems. We conclude with discussion of the obtained results and outline possible generalizations of the developed formalism.

## Results

### Envelope dyadic Green’s function technique

In order to investigate the propagation of the EMFSVA in a dispersive medium, we consider the Maxwell equations for the time-dependent electromagnetic fields and sources written as follows

1 $$\nabla \cdot {\bf{D}}={\rho }^{E},\,\nabla \cdot {\bf{B}}={\rho }^{M},$$

2 $$\nabla \times {\bf{E}}=-\,{\partial }_{t}{\bf{B}}-{{\bf{j}}}^{M},\,\nabla \times {\bf{H}}={\partial }_{t}{\bf{D}}+{{\bf{j}}}^{E},$$

where the vectors ${\bf{E}}$, ${\bf{H}}$, ${\bf{D}}$, and ${\bf{B}}$ are the electric field, the magnetic field, the electric displacement, and the magnetic flux density, respectively, and ${\partial }_{t}=\frac{\partial }{\partial t}$. In Eqs. (1) and (2), ${\rho }^{E(M)}$ and ${{\bf{j}}}^{E(M)}$, the electric (magnetic) charge and current densities, respectively, are related by the continuity equation, $\nabla \cdot {{\bf{j}}}^{E(M)}+{\partial }_{t}{\rho }^{E(M)}=0$. The electromagnetic constitutive equation is given by

3 $${\boldsymbol{\Xi }}=\bar{\bar{\zeta }}\cdot {\bf{f}},$$

where the dyadic integro-differential operator $\bar{\bar{\zeta }}=\bar{\bar{\varepsilon }}$ ($\bar{\bar{\mu }}$) represents the dispersive anisotropic permittivity (permeability) of the material, which relates ${\boldsymbol{\Xi }}={\bf{D}}\,({\bf{B}})$ to ${\bf{f}}={\bf{E}}\,({\bf{H}})$.

The time-dependent electromagnetic field can be expanded into the monochromatic spectral components as follows

4 $${\bf{f}}(t)=\frac{1}{2\pi }\,{\int }_{-\infty }^{+\infty }\,{{\bf{f}}}_{\omega }(\omega ){e}^{-i\omega t}d\omega .$$

On the other hand, considering the time-harmonic fields with SVAs, it is acceptable that the most spectral energy is concentrated in a narrow band Δ$\omega$ around ${\omega }_{0}$, the carrier frequency, with $\Delta \omega \ll {\omega }_{0}$. Thus, we can define the EMFSVA, ${{\bf{f}}}_{m}(t)$, as follows

5 $${\bf{f}}(t)=\frac{1}{2}{{\bf{f}}}_{m}(t){e}^{-i{\omega }_{0}t}+{\rm{c}}.{\rm{c}}.,$$

6 $${{\bf{f}}}_{m}(t)=\frac{1}{\pi }\,{\int }_{-\Delta \omega /2}^{+\Delta \omega /2}\,{{\bf{f}}}_{\omega }({\omega }_{0}+\Omega ){e}^{-i\Omega t}d\Omega ,$$

where c.c. is the abbreviation for the complex conjugate of the previous term and $\Omega =\omega -{\omega }_{0}$. We also assume that ${\bar{\bar{\zeta }}}_{\omega }$, the Fourier transform of $\bar{\bar{\zeta }}$, has appreciably smooth behavior in the narrow band Δ$\omega$, which is a reasonable assumption for applications of our interest. So, expanding ${\bar{\bar{\zeta }}}_{\omega }$ around ${\omega }_{0}$ and keeping only the two first terms, we obtain

7 $${\bar{\bar{\zeta }}}_{\omega }={\bar{\bar{\zeta }}}_{\omega }{|}_{{\omega }_{0}}+({\partial }_{\omega }{\bar{\bar{\zeta }}}_{\omega }){|}_{{\omega }_{0}}\Omega ,$$

where ${\partial }_{\omega }=\frac{\partial }{\partial \omega }$ and ${|}_{{\omega }_{0}}$ denotes that the related quantity is computed at ${\omega }_{0}$. Using Eqs. (5–7), we obtain ${\partial }_{t}{\boldsymbol{\Xi }}$ as follows

8 $${\partial }_{t}{\boldsymbol{\Xi }}={e}^{-i{\omega }_{0}t}({\partial }_{t}-i{\omega }_{0}){{\boldsymbol{\Xi }}}_{m}(t),$$

9 $${{\boldsymbol{\Xi }}}_{m}(t)=({\bar{\bar{\zeta }}}_{\omega }{|}_{{\omega }_{0}}+i({\partial }_{\omega }{\bar{\bar{\zeta }}}_{\omega }){|}_{{\omega }_{0}}{\partial }_{t})\cdot {{\bf{f}}}_{m}(t)\equiv {\bar{\bar{\zeta }}}_{m}\cdot {{\bf{f}}}_{m}(t),$$

where the dyadic differential operator ${\bar{\bar{\zeta }}}_{m}$ is ${\bar{\bar{\zeta }}}_{m}={\bar{\bar{\zeta }}}_{\omega }{|}_{{\omega }_{0}}+i({\partial }_{\omega }{\bar{\bar{\zeta }}}_{\omega }){|}_{{\omega }_{0}}{\partial }_{t}$, and we have to ignore the second and higher orders of ${\partial }_{t}$ in expressions involving this operator. With having these tools at hand, we proceed to the Green’s function technique to solve the Maxwell equations written in terms of the EMFSVA.

Starting from Eqs. (1) and (2) for the time-dependent electromagnetic fields and sources and using Eqs. (3–9), the Maxwell 6-vector equations for the EMFSVA assume the following operator form:

10 $$\hat{{\bf{O}}}\cdot {{\bf{F}}}_{m}=-\,i{{\bf{J}}}_{m}.$$

In the above equation, ${{\bf{F}}}_{m}$, the SVA of the 6-vector field, ${{\bf{J}}}_{m}$, the 6-vector current density, and $\hat{{\bf{O}}}$, the electromagnetic operator, are given by

11 $${{\bf{F}}}_{m}\dot{=}[\begin{array}{l}{{\bf{E}}}_{m}\\ {{\bf{H}}}_{m}\end{array}],\,{{\bf{J}}}_{m}\dot{=}[\begin{array}{l}{{\bf{j}}}_{m}^{E}\\ {{\bf{j}}}_{m}^{M}\end{array}],\,\hat{{\bf{O}}}\dot{=}i[\begin{array}{cc}({\partial }_{t}-i{\omega }_{0}){\bar{\bar{\varepsilon }}}_{m} & -\,\nabla \times \bar{\bar{I}}\\ \nabla \times \bar{\bar{I}} & ({\partial }_{t}-i{\omega }_{0}){\bar{\bar{\mu }}}_{m}\end{array}],$$

where $\bar{\bar{I}}$ is the identity dyadic. In Eq. (11), the differential operators $({\partial }_{t}-i{\omega }_{0}){\bar{\bar{\zeta }}}_{m}$ are expanded as

12 $$({\partial }_{t}-i{\omega }_{0}){\bar{\bar{\zeta }}}_{m}=-\,i{\omega }_{0}{\bar{\bar{\zeta }}}_{\omega }{|}_{{\omega }_{0}}+{\partial }_{\omega }(\omega {\bar{\bar{\zeta }}}_{\omega }){|}_{{\omega }_{0}}{\partial }_{t},$$

with the second order derivative term ignored, in line with the assumption of the SVA. We may introduce the envelope 6-dyadic Green’s function (EDGF), $\overline{\bar{{\bf{G}}}}({\bf{r}},t;{\bf{r}}^{\prime} ,t^{\prime} )$, for Eq. (10) such that

13 $$\hat{{\bf{O}}}\cdot \bar{\bar{{\bf{G}}}}=-\,i\delta ({\bf{r}}-{\bf{r}}^{\prime} )\tilde{\delta }(t-t^{\prime} )\bar{\bar{{\bf{I}}}},$$

where $\overline{\bar{{\bf{I}}}}$ is the identity 6-dyadic, and $\tilde{\delta }(\cdot )$, similar in role to the Dirac delta $\delta (\cdot )$, emphasizes that we consider the slowly varying sources and fields. Respectively, $\tilde{\delta }(t)$ has a finite duration and $|\tilde{\delta }(t)| < \infty$. Equation (13) means that the EDGF is obtained by inverting the operator $\hat{{\bf{O}}}$.

It is more convenient to work in the Fourier domain when the material parameters are uniform in both space and time. In this case, $\overline{\bar{{\bf{G}}}}({\bf{r}},t;{\bf{r}}^{\prime} ,t^{\prime} )\equiv \overline{\bar{{\bf{G}}}}({\bf{r}}-{\bf{r}}^{\prime} ,t-t^{\prime} )$, and we can define $\overline{\bar{{\bf{g}}}}({\bf{k}},\Omega )$, the Fourier transform of the EDGF, by the following relation

14 $$\bar{\bar{{\bf{G}}}}({\bf{R}},\tau )=\frac{1}{{(2\pi )}^{4}}\,{\int }^{}\,d{\bf{k}}\,{\int }_{-\Delta \omega /2}^{+\Delta \omega /2}\,d\Omega \,\bar{\bar{{\bf{g}}}}({\bf{k}},\Omega ){e}^{i({\bf{k}}\cdot {\bf{R}}-\Omega \tau )},$$

where ${\bf{R}}={\bf{r}}-{\bf{r}}^{\prime}$ and $\tau =t-t^{\prime}$. Recalling Eq. (13) and using that, in the Fourier space, ${\partial }_{t}=-\,i\Omega$ and $\nabla =i{\bf{k}}$, where ${\bf{k}}$ is the wave vector, we find that the 6-dyadic equation for $\overline{\bar{{\bf{g}}}}$ satisfies

15 $$\hat{{\bf{O}}}\cdot \bar{\bar{{\bf{g}}}}=-\,i\bar{\bar{{\bf{I}}}},\,\hat{{\bf{O}}}\dot{=}[\begin{array}{cc}{D}_{\Omega }{\bar{\bar{\varepsilon }}}_{\omega } & {\bf{k}}\times \bar{\bar{I}}\\ -{\bf{k}}\times \bar{\bar{I}} & {D}_{\Omega }{\bar{\bar{\mu }}}_{\omega }\end{array}],$$

where we have used operator-like notation for ${D}_{\Omega }$ such that

16 $${D}_{\Omega }{\bar{\bar{\zeta }}}_{\omega }\equiv {\omega }_{0}{\bar{\bar{\zeta }}}_{\omega }{|}_{{\omega }_{0}}+\Omega \,{\partial }_{\omega }(\omega {\bar{\bar{\zeta }}}_{\omega }){|}_{{\omega }_{0}},$$

and the fact that $\tilde{\delta }(\tau )={(2\pi )}^{-1}\,{\int }_{-\Delta \omega /2}^{+\Delta \omega /2}\,{e}^{-i\Omega \tau }\,d\Omega$.

Knowing the dispersive constitutive parameters of the medium and inverting the operator $\hat{{\bf{O}}}$ in Eq. (15), we can obtain $\overline{\bar{{\bf{g}}}}$ and, in turn, the EDGF. Afterward, for any given forms of the 6-vector source functions ${{\bf{J}}}_{m}$ with the frequency spectra fitting the interval $\Omega \in [-\frac{\Delta \omega }{2};+\,\frac{\Delta \omega }{2}]$, the components of the 6-vector ${{\bf{F}}}_{m}$ are obtained with the help of Kotelnikov’s theorem^52^ (see Methods) as follows

17 $${{\bf{F}}}_{m}({\bf{r}},t)=\frac{2\pi }{\Delta \omega }\,\mathop{\sum }\limits_{n=-\infty }^{+\infty }\,{\int }^{}\,d{\bf{r}}^{\prime} \,\bar{\bar{{\bf{G}}}}({\bf{r}}-{\bf{r}}^{\prime} ,t-{t}_{n})\cdot {{\bf{J}}}_{m}({\bf{r}}^{\prime} ,{t}_{n}),$$

where ${t}_{n}=\frac{2\pi n}{\Delta \omega }$. In the next section, we shall find the matrix elements of the EDGF for uniaxial anisotropic media.

### EDGF matrix elements

Here, we apply the EDGF technique for reciprocal uniaxial media to obtain the matrix elements. For such media we can write

18 $${\bar{\bar{\zeta }}}_{\omega }={\zeta }_{t}{\bar{\bar{I}}}_{t}+{\zeta }_{z}{\bar{\bar{I}}}_{z}$$

with ${\bar{\bar{I}}}_{t}=\hat{{\bf{x}}}\hat{{\bf{x}}}+\hat{{\bf{y}}}\hat{{\bf{y}}}$ and ${\bar{\bar{I}}}_{z}=\hat{{\bf{z}}}\hat{{\bf{z}}}$ being the projection dyadics in the transverse and axial (the main axis) directions, respectively, along which the electromagnetic responses of the medium are different. In order to obtain the EDGF of the uniaxial medium, we impose this property of constitutive parameters to decompose the operator $\hat{{\bf{O}}}$ in Eq. (15) as19where $\overline{\bar{0}}$ is the null dyadic. When writing the matrix elements in Eq. (19), we keep in mind that ${\bf{k}}\times \bar{\bar{I}}=({{\bf{k}}}_{t}+{{\bf{k}}}_{z})\times ({\bar{\bar{I}}}_{t}+{\bar{\bar{I}}}_{z})$, where ${{\bf{k}}}_{t}={\bar{\bar{I}}}_{t}\cdot {\bf{k}}$ and ${{\bf{k}}}_{z}={\bar{\bar{I}}}_{z}\cdot {\bf{k}}$. Next, it is more convenient if we replace the second row with the third one and also the second column with the third one in the matrix representation of $\hat{{\bf{O}}}$ in Eq. (19) so that the tangential and axial components of the constitutive parameters of the medium take place in separate blocks. When doing this, it is also necessary to respectively rearrange the dyadic components of $\overline{\bar{{\bf{g}}}}$ and $\overline{\bar{{\bf{I}}}}$ in Eq. (15). After doing this and taking into account that ${{\bf{k}}}_{t}\times {\bar{\bar{I}}}_{t}={\bar{\bar{I}}}_{z}\times {{\bf{k}}}_{t}$, the EDGF is expressed through the inverse of the reordered matrix of $\hat{{\bf{O}}}$ as follows20

Representation (20) allows for a straightforward splitting of the fields into a pair of orthogonal polarizations: The transverse-electric (TE or *s*-) polarization with vanishing axial component of the electric field, and the transverse-magnetic (TM or *p*-) polarization with vanishing axial component of the magnetic field. In order to perform such splitting, ${\bar{\bar{I}}}_{t}$ in Eq. (20) is expanded as $${\bar{\bar{I}}}_{t}=\frac{{{\bf{k}}}_{t}{{\bf{k}}}_{t}}{{k}_{t}^{2}}+{\bar{\bar{I}}}_{z}\begin{array}{c}\times \\ \times \end{array}\frac{{{\bf{k}}}_{t}{{\bf{k}}}_{t}}{{k}_{t}^{2}}$$, where $$\begin{array}{c}\times \\ \times \end{array}$$ denotes the dyadic double cross product: $${\bf{a}}{\bf{b}}\begin{array}{c}\times \\ \times \end{array}{\bf{c}}{\bf{d}}={\bf{a}}\times {\bf{c}}\,{\bf{b}}\times {\bf{d}}$$ and ${k}_{t}=|{{\bf{k}}}_{t}|$. Then, after a rather tedious but straightforward dyadic algebra, the matrix in Eq. (20) can be inverted and the following result obtained:

21 $${\overline{\bar{{\bf{g}}}}}^{tt}=[\begin{array}{cc}{g}_{ee}^{tt,p}\frac{{{\bf{k}}}_{t}{{\bf{k}}}_{t}}{{k}_{t}^{2}} & {g}_{em}^{tt,p}\frac{{{\bf{k}}}_{t}\hat{{\bf{z}}}\times {{\bf{k}}}_{t}}{{k}_{t}^{2}}\\ {g}_{em}^{tt,p}\frac{\hat{{\bf{z}}}\times {{\bf{k}}}_{t}{{\bf{k}}}_{t}}{{k}_{t}^{2}} & {g}_{mm}^{tt,p}\frac{\hat{{\bf{z}}}\hat{{\bf{z}}}\begin{array}{c}\times \\ \times \end{array}{{\bf{k}}}_{t}{{\bf{k}}}_{t}}{{k}_{t}^{2}}\end{array}]+[\begin{array}{cc}{g}_{ee}^{tt,s}\frac{\hat{{\bf{z}}}\hat{{\bf{z}}}\begin{array}{c}\times \\ \times \end{array}{{\bf{k}}}_{t}{{\bf{k}}}_{t}}{{k}_{t}^{2}} & {g}_{em}^{tt,s}\frac{\hat{{\bf{z}}}\times {{\bf{k}}}_{t}{{\bf{k}}}_{t}}{{k}_{t}^{2}}\\ {g}_{em}^{tt,s}\frac{{{\bf{k}}}_{t}\hat{{\bf{z}}}\times {{\bf{k}}}_{t}}{{k}_{t}^{2}} & {g}_{mm}^{tt,s}\frac{{{\bf{k}}}_{t}{{\bf{k}}}_{t}}{{k}_{t}^{2}}\end{array}],$$

22 $${\overline{\bar{{\bf{g}}}}}^{tz}={({\overline{\bar{{\bf{g}}}}}^{zt})}^{T}=[\begin{array}{cc}{g}_{ee}^{tz,p}\frac{{{\bf{k}}}_{t}\hat{{\bf{z}}}}{{k}_{t}} & {g}_{em}^{tz,s}\frac{\hat{{\bf{z}}}\times {{\bf{k}}}_{t}\hat{{\bf{z}}}}{{k}_{t}}\\ {g}_{me}^{tz,p}\frac{\hat{{\bf{z}}}\times {{\bf{k}}}_{t}\hat{{\bf{z}}}}{{k}_{t}} & {g}_{mm}^{tz,s}\frac{{{\bf{k}}}_{t}\hat{{\bf{z}}}}{{k}_{t}}\end{array}],$$

23 $${\bar{\bar{g}}}^{zz}=[\begin{array}{cc}{g}_{ee}^{zz,p}{\bar{\bar{I}}}_{z} & \overline{\bar{0}}\\ \overline{\bar{0}} & {g}_{mm}^{zz,s}{\bar{\bar{I}}}_{z}\end{array}],$$

where the 12 independent non-vanishing components of $\overline{\bar{{\bf{g}}}}$ are expressed as follows (here and thereafter we use shorthand notation $\alpha [\beta ]$ for respective selection of either $\alpha$ or $\beta$, where $\alpha$ and $\beta$ are isolated terms or groups of indices):

24 $$\begin{array}{l}{g}_{ee[mm]}^{tt,p[s]}=-\,i\frac{{d}_{t}^{m[e]}-{k}_{t}^{2}/{d}_{z}^{e[m]}}{{\kappa }_{p[s]}^{2}-{k}_{z}^{2}},\,{g}_{ee[mm]}^{tt,s[p]}=-\,\frac{i{d}_{t}^{m[e]}}{{\kappa }_{s[p]}^{2}-{k}_{z}^{2}},\,{g}_{em}^{tt,s[p]}=[\,-\,]\frac{i{k}_{z}}{{\kappa }_{s[p]}^{2}-{k}_{z}^{2}},\\ {g}_{ee[mm]}^{tz,p[s]}=\frac{i{k}_{t}{k}_{z}/{d}_{z}^{e[m]}}{{\kappa }_{p[s]}^{2}-{k}_{z}^{2}},\,{g}_{me[em]}^{tz,p[s]}=[\,-\,]\frac{i{k}_{t}{d}_{t}^{e[m]}/{d}_{z}^{e[m]}}{{\kappa }_{p[s]}^{2}-{k}_{z}^{2}},\,{g}_{ee[mm]}^{zz,p[s]}=-\,i\frac{({d}_{t}^{e}{d}_{t}^{m}-{k}_{z}^{2})/{d}_{z}^{e[m]}}{{\kappa }_{p[s]}^{2}-{k}_{z}^{2}},\end{array}$$

where, from Eq. (16),

25 $$\begin{array}{l}{d}_{l}^{e[m]}={a}_{l}^{e[m]}+{b}_{l}^{e[m]}\Omega ,\,{a}_{l}^{e[m]}={\omega }_{0}{\varepsilon }_{l}[{\mu }_{l}]{|}_{{\omega }_{0}},\,{b}_{l}^{e[m]}={\partial }_{\omega }(\omega {\varepsilon }_{l}[{\mu }_{l}]){|}_{{\omega }_{0}},\end{array}$$

where the index $l$ is either $t$ or $z$, and

26 $${\kappa }_{p[s]}=\sqrt{{d}_{t}^{e}{d}_{t}^{m}-({d}_{t}^{e[m]}/{d}_{z}^{e[m]}){k}_{t}^{2}},\,{\rm{Im}}({\kappa }_{p[s]})\ge 0.$$

As can be seen from Eq. (24), there are two poles in ${k}_{z}$ for each polarization: $\pm {\kappa }_{p}$ for the TM case and, similarly, $\pm {\kappa }_{s}$ for the TE one. Physically, the poles ${k}_{z}=+\,{\kappa }_{p[s]}$ correspond to the waves propagating in the halfspace $z-z^{\prime}  > 0$, and the poles with ${k}_{z}=-\,{\kappa }_{p[s]}$ correspond to the waves propagating at $z-z^{\prime}  < 0$.

Based on Eq. (24), we can sort out the components of $\overline{\bar{{\bf{g}}}}$ into two groups which correspond to the waves of $p$- and $s$-polarization

27 $$\overline{\bar{{\bf{g}}}}=\frac{{\overline{\bar{{\bf{N}}}}}_{p}}{{\kappa }_{p}^{2}-{k}_{z}^{2}}+\frac{{\overline{\bar{{\bf{N}}}}}_{s}}{{\kappa }_{s}^{2}-{k}_{z}^{2}},$$

where ${\overline{\bar{{\bf{N}}}}}_{p[s]}$ are formed by the corresponding terms in the numerators of Eq. (24). Respectively, when taking the inverse Fourier transform of $\overline{\bar{{\bf{g}}}}$ as given by Eq. (14) and considering the integral over $d{k}_{z}$, we get two categories of residues

28 $$\frac{1}{2\pi }\,{\int }_{-\infty }^{\infty }\,d{k}_{z}\,\overline{\bar{{\bf{g}}}}\,{e}^{i{k}_{z}Z}=-\,\sum _{\gamma =p,s}\,\frac{i}{2{\kappa }_{\gamma }}{\overline{\bar{{\bf{N}}}}}_{\gamma }{|}_{{k}_{z}={\rm{sgn}}(Z){\kappa }_{\gamma }}{e}^{i{\kappa }_{\gamma }|Z|}=\sum _{\gamma =p,s}\,{\overline{\bar{{\bf{A}}}}}_{\gamma ,{\rm{sgn}}(Z)}{e}^{i{\kappa }_{\gamma }|Z|},$$

where $Z=z-z^{\prime}$, and ${\rm{sgn}}(Z)=\pm \,1$ is the sign of *Z*.

Next, before taking the integral over $d\Omega$, we recall the approximation of the SVA, and expand ${\overline{\bar{{\bf{A}}}}}_{\gamma ,\pm 1}$ and ${\kappa }_{\gamma }$ around the point $\Omega =0$ and ignore $O({\Omega }^{2})$ terms so that

29 $${\overline{\bar{{\bf{A}}}}}_{\gamma ,\pm 1}={\overline{\bar{{\bf{A}}}}}_{\gamma ,\pm 1}{|}_{\Omega =0}+\Omega {\frac{\partial {\overline{\bar{{\bf{A}}}}}_{\gamma ,\pm 1}}{\partial \Omega }|}_{\Omega =0}\equiv {\overline{\bar{{\bf{C}}}}}_{\gamma ,\pm 1}+\Omega \,{\overline{\bar{{\bf{D}}}}}_{\gamma ,\pm 1},$$

30 $${\kappa }_{\gamma }={\kappa }_{\gamma }{|}_{\Omega =0}+\Omega {\frac{\partial {\kappa }_{\gamma }}{\partial \Omega }|}_{\Omega =0}\equiv {\kappa }_{0}^{\gamma }+\Omega /{V}_{g}^{\gamma },$$

where the expressions for the components of ${\overline{\bar{{\bf{C}}}}}_{\gamma ,\pm 1}={\overline{\bar{{\bf{A}}}}}_{\gamma ,\pm 1}{|}_{\Omega =0}$ and ${\overline{\bar{{\bf{D}}}}}_{\gamma ,\pm 1}=\partial {\overline{\bar{{\bf{A}}}}}_{\gamma ,\pm 1}/\partial \Omega \,{|}_{\Omega =0}$ are given in Methods, and for the propagation factor ${\kappa }_{0}^{\gamma }={\kappa }_{\gamma }{|}_{\Omega =0}$ and the complex group velocity ${V}_{g}^{\gamma }={(\partial {\kappa }_{\gamma }/\partial \Omega )}_{\Omega =0}^{-1}$ we obtain

31 $${\kappa }_{0}^{p[s]}=\sqrt{{a}_{t}^{e}{a}_{t}^{m}-\frac{{a}_{t}^{e[m]}}{{a}_{z}^{e[m]}}{k}_{t}^{2}},$$

32 $${V}_{g}^{p[s]}=2{\kappa }_{0}^{p[s]}/(({a}_{t}^{e}{b}_{t}^{m}+{a}_{t}^{m}{b}_{t}^{e})-\frac{{k}_{t}^{2}}{{a}_{z}^{{e[m]}^{2}}}({b}_{t}^{e[m]}{a}_{z}^{e[m]}-{a}_{t}^{e[m]}{b}_{z}^{e[m]})),$$

for the *p*- [*s*-] polarization. It should be noted that ignoring the second order terms in Eqs. (29) and (30) is reasonable for the frequency intervals of our interest, where the group velocity dispersion effects are relatively weak. In Methods, we discuss this with more detail and also present a closer look at the group velocity for a propagating envelope in an active and dispersive medium.

With these approximations at hand, when performing the integration over $\Omega$ we obtain

33 $$\begin{array}{c}\frac{1}{2\pi }\,{\int }_{-\Delta \omega /2}^{+\Delta \omega /2}\,d\Omega \,\sum _{\gamma =p,s}\,({\overline{\bar{{\bf{C}}}}}_{\gamma ,{\rm{sgn}}(Z)}+\Omega \,{\overline{\bar{{\bf{D}}}}}_{\gamma ,{\rm{sgn}}(Z)}){e}^{i{\kappa }_{0}^{\gamma }|Z|-i\Omega {\tau }_{g}^{\gamma }}\\ \,=\,\frac{\Delta \omega }{2\pi }\,\sum _{\gamma =p,s}\,{e}^{i{\kappa }_{0}^{\gamma }|Z|}[{\overline{\bar{{\bf{C}}}}}_{\gamma ,{\rm{sgn}}(Z)}\,{j}_{0}(\Delta \omega {\tau }_{g}^{\gamma }/2)-\frac{i\Delta \omega }{2}{\overline{\bar{{\bf{D}}}}}_{\gamma ,{\rm{sgn}}(Z)}\,{j}_{1}(\Delta \omega {\tau }_{g}^{\gamma }/2)],\end{array}$$

where ${\tau }_{g}^{\gamma }=\tau -|Z|/{V}_{g}^{\gamma }$, and ${j}_{n}(x)$ denotes the spherical Bessel function of the first kind and the *n*-th order. Therefore, from Eq. (14), we obtain the EDGF, $\overline{\bar{{\bf{G}}}}({\bf{R}},\tau )$, in the following form

34 $$\overline{\bar{{\bf{G}}}}=\frac{\Delta \omega }{{(2\pi )}^{3}}\,\sum _{\gamma =p,s}\,{\int }^{}\,d{{\bf{k}}}_{t}\,{e}^{i({{\bf{k}}}_{t}\cdot {\bf{R}}+{\kappa }_{0}^{\gamma }|\hat{{\bf{z}}}\cdot {\bf{R}}|)}{\overline{\bar{{\bf{T}}}}}_{\gamma ,{\rm{sgn}}(\hat{{\bf{z}}}\cdot {\bf{R}})}(\frac{\Delta \omega }{2}(\tau -\frac{|\hat{{\bf{z}}}\cdot {\bf{R}}|}{{V}_{g}^{\gamma }})),$$

where

35 $${\overline{\bar{{\bf{T}}}}}_{\gamma ,\pm 1}(x)={\overline{\bar{{\bf{C}}}}}_{\gamma ,\pm 1}\,{j}_{0}(x)-\frac{i\Delta \omega }{2}{\overline{\bar{{\bf{D}}}}}_{\gamma ,\pm 1}\,{j}_{1}(x).$$

As can be inferred from Eqs. (34) and (35), besides the usual effect of the imaginary part of the propagation factor, ${\rm{Im}}({\kappa }_{0}^{\gamma })$, the imaginary part of the complex group velocity, ${\rm{Im}}({V}_{g}^{\gamma })$, is responsible for an additional change in the envelope’s magnitude during propagation.

In some special cases, the remaining integration over $d{{\bf{k}}}_{t}$ in Eq. (34) can be performed analytically. In the following subsection, we consider such a special case of paraxial propagation. The representation (34) is most suitable for the calculation of fields of sources with a known spatial spectrum in the transverse plane, e.g. in near-field radiative heat transfer problems. Considering arbitrary source vectors and using Eqs. (17) and (34), we can investigate the propagation of the EMFSVA in any unbounded anisotropic dispersive media.

The present formalism can be extended to contain interface effects, which will make it applicable to investigate the propagation of the EMFSVA via multilayer media. In what follows, we consider a special case of such media when the neighboring layers are (approximately) impedance matched.

### EDGF for paraxial propagation

Let us consider the case when the envelope propagation happens dominantly along the anisotropy axis. This case is typical for extremely anisotropic uniaxial MM in which $|{\varepsilon }_{z}|\gg |{\varepsilon }_{t}|$ and (or) $|{\mu }_{z}|\gg |{\mu }_{t}|$. Indeed, the propagation factor ${\kappa }_{0}^{\gamma }$ from Eq. (31) can be expressed as

36 $${\kappa }_{0}^{p[s]}={\kappa }_{a}\sqrt{1-\frac{{a}_{t}^{e[m]}{k}_{t}^{2}}{{a}_{z}^{e[m]}{\kappa }_{a}^{2}}},$$

where ${\kappa }_{a}=\sqrt{{a}_{t}^{e}{a}_{t}^{m}}$. When $|{a}_{z}^{e[m]}{k}_{t}^{2}|\ll |{a}_{z}^{e[m]}{\kappa }_{a}^{2}|$, we can expand ${\kappa }_{0}^{\gamma }$ as follows

37 $${\kappa }_{0}^{p[s]}\approx {\kappa }_{a}-\frac{1}{2}\frac{{a}_{t}^{e[m]}{k}_{t}^{2}}{{a}_{z}^{e[m]}{\kappa }_{a}}.$$

On the other hand, from Eq. (24) it is seen that in this case the ${\overline{\bar{{\bf{g}}}}}_{tt}$ component dominates over the ${\overline{\bar{{\bf{g}}}}}_{tz}$, ${\overline{\bar{{\bf{g}}}}}_{zt}$ and ${\overline{\bar{{\bf{g}}}}}_{zz}$ components, so that the EDGF is dominantly transverse which implies that the wave energy propagates dominantly along the *z*-axis.

Therefore, when calculating the integral over $d{{\bf{k}}}_{t}$ in Eq. (34), we will not make a big mistake if we evaluate the ${\overline{\bar{{\bf{T}}}}}_{\gamma ,\pm 1}$ term of Eq. (34) at ${k}_{t}\to 0$ while using the expansion (37) in the exponential terms. In this approximation, the integration over $d{{\bf{k}}}_{t}$ can be performed analytically, which results in the following expression for the $tt$-block of the paraxial EDGF:

38 $${\overline{\bar{{\bf{g}}}}}_{a}^{tt}(x-x^{\prime} ,y-y^{\prime} ,\pm \,|z-z^{\prime} |,t-t^{\prime} )=\frac{\Delta \omega }{{(2\pi )}^{2}}\,\sum _{\gamma =p,s}\,{\int }_{-\infty }^{{\xi }_{\gamma }}\,d\xi {\overline{\bar{{\bf{t}}}}}_{\gamma \pm }\frac{{e}^{i{\kappa }_{a}(|z-z^{\prime} |+\frac{{(x-x^{\prime} )}^{2}+{(y-y^{\prime} )}^{2}}{2\xi })}}{2\xi },$$

where $${\xi }_{p[s]}=\frac{{a}_{t}^{e[m]}}{{a}_{z}^{e[m]}}|z-z^{\prime} |$$, and

39 $${\overline{\bar{{\bf{t}}}}}_{s\pm }=[\begin{array}{cc}-\frac{{a}_{t}^{m}\hat{{\bf{z}}}\hat{{\bf{z}}}\begin{array}{c}\times \\ \times \end{array}{\nabla }_{t}{\nabla }_{t}}{2{\kappa }_{a}} & \pm \frac{\hat{{\bf{z}}}\times {\nabla }_{t}{\nabla }_{t}}{2}\\ \mp \frac{{\nabla }_{t}{\nabla }_{t}\times \hat{{\bf{z}}}}{2} & -\frac{{a}_{t}^{e}{\nabla }_{t}{\nabla }_{t}}{2{\kappa }_{a}}\end{array}]\,{j}_{0}(\tau )-\,\frac{i\Delta \omega }{2}(\frac{{b}_{t}^{m}}{{a}_{t}^{m}}-\frac{{b}_{t}^{e}}{{a}_{t}^{e}})[\begin{array}{cc}-\frac{{a}_{t}^{m}\hat{{\bf{z}}}\hat{{\bf{z}}}\begin{array}{c}\times \\ \times \end{array}{\nabla }_{t}{\nabla }_{t}}{4{\kappa }_{a}} & \overline{\bar{0}}\\ \overline{\bar{0}} & \frac{{a}_{t}^{e}{\nabla }_{t}{\nabla }_{t}}{4{\kappa }_{a}}\end{array}]\,{j}_{1}(\tau ),$$

40 $${\overline{\bar{{\bf{t}}}}}_{p\pm }=[\begin{array}{cc}-\frac{{a}_{t}^{m}{\nabla }_{t}{\nabla }_{t}}{2{\kappa }_{a}} & \pm \frac{{\nabla }_{t}{\nabla }_{t}\times \hat{{\bf{z}}}}{2}\\ \mp \frac{\hat{{\bf{z}}}\times {\nabla }_{t}{\nabla }_{t}}{2} & -\frac{{a}_{t}^{e}\hat{{\bf{z}}}\hat{{\bf{z}}}\begin{array}{c}\times \\ \times \end{array}{\nabla }_{t}{\nabla }_{t}}{2{\kappa }_{a}}\end{array}]\,{j}_{0}(\tau )-\,\frac{i\Delta \omega }{2}(\frac{{b}_{t}^{m}}{{a}_{t}^{m}}-\frac{{b}_{t}^{e}}{{a}_{t}^{e}})[\begin{array}{cc}-\frac{{a}_{t}^{m}{\nabla }_{t}{\nabla }_{t}}{4{\kappa }_{a}} & \overline{\bar{0}}\\ \overline{\bar{0}} & \frac{{a}_{t}^{e}\hat{{\bf{z}}}\hat{{\bf{z}}}\begin{array}{c}\times \\ \times \end{array}{\nabla }_{t}{\nabla }_{t}}{4{\kappa }_{a}}\end{array}]\,{j}_{1}(\tau ),$$

with ${\nabla }_{t}=\hat{{\bf{x}}}(\partial /\partial x)+\hat{{\bf{y}}}(\partial /\partial y)$, $\tau =\frac{\Delta \omega }{2}(t-t^{\prime} -|z-z^{\prime} |/{V}_{g}^{a})$, and ${V}_{g}^{a}=2{\kappa }_{a}/({a}_{t}^{e}{b}_{t}^{m}+{a}_{t}^{m}{b}_{t}^{e})$. The integral over $d\xi$ in Eq. (38) can be taken in a closed form after the dyadic differential operators ${\overline{\bar{{\bf{t}}}}}_{\gamma \pm }$ have acted on the exponential term. The integral representation (38) is especially handy when taking the convolution integral in Eq. (17) with the source currents having Gaussian profiles in the $xy$-plane.

### Propagation through impedance matched MM layers

For the sake of this section, we apply the developed formalism for multilayer uniaxial MMs in which layers are approximately impedance matched. The anisotropy axis is along the $z$-axis and is the same in all layers. The interfaces of the layers are perpendicular to the $z$-axis and are located at planes $z={z}_{l}$, where $l$ is the layer index. Here, we are interested only in the paraxial propagation.

As the source of excitation, we consider oscillating surface electric current distributed in the plane $z=z^{\prime}$ with some amplitude profile along the $x$-direction. The plane $z=z^{\prime}$ happens to be inside one of the layers (which we will call the 0-th layer) located at $z\in ({z}_{0},{z}_{1})$. In the $y$-direction, the source current is uniform. The source current density is oscillating in time with the carrier frequency ${\omega }_{0}$ and has the SVA envelope of oscillations, ${{\bf{j}}}_{m}^{E}$, defined as

41 $${{\bf{j}}}_{m}^{E}=\sum _{\gamma =p,s}\,{J}_{\gamma }{\hat{{\bf{u}}}}_{\gamma }{e}^{-\frac{{x}^{2}}{2{\sigma }_{\gamma }^{2}}-\frac{{t}^{2}}{2{\sigma }_{t}^{2}}}\delta (z-z^{\prime} ),$$

where ${J}_{\gamma }{\hat{{\bf{u}}}}_{\gamma }$ with $\gamma =p,s$, determines the initial vectorial amplitudes of the *s*- and *p*-polarized components of the surface current (here, ${\hat{{\bf{u}}}}_{p}=\hat{{\bf{x}}}$ and ${\hat{{\bf{u}}}}_{s}=\hat{{\bf{y}}}$), ${\sigma }_{p[s]}$ defines the characteristic width of the amplitude profiles in $x$, separately for the two polarizations, and where we assume that ${\sigma }_{t}$, the envelope duration in time, is such that $\frac{\Delta \omega }{2\pi }{\sigma }_{t}\gtrsim 1$. Under this condition, practically all source spectral power is concentrated within the frequency interval of width $\Delta \omega$. Thus, the SVA of the 6-vector source current density reads

42 $${{\bf{J}}}_{m}(x,z,t)={{\bf{J}}}_{0}(x,t)\delta (z-z^{\prime} ),\,{{\bf{J}}}_{0}(x,t)=\sum _{\gamma =p,s}\,[\begin{array}{c}{J}_{\gamma }{\hat{{\bf{u}}}}_{\gamma }\\ 0\end{array}]{e}^{-\frac{{x}^{2}}{2{\sigma }_{\gamma }^{2}}-\frac{{t}^{2}}{2{\sigma }_{t}^{2}}}.$$

Such a source creates the electromagnetic field in the 0-th layer which propagates in both $z > z^{\prime}$ and $z < z^{\prime}$ directions. When this field reaches the interface $z={z}_{1}$ between the 0th and the next layer, it excites the fields in the next layer, and so on. If the characteristic wave impedances of the neighboring layers are mismatched, the reflected field will also appear, which can propagate to the other interface, be partially reflected again, etc.

We reserve the study of such multiple reflections in the EDGF context for a future work. Here, we assume that the neighboring layers are approximately impedance matched at the frequencies close to ${\omega }_{0}$, and the reflections may be neglected. Note that this does not mean that the layers must be made of the same materials, or that the materials must have the same dispersion. The impedance match condition for the case of the paraxial propagation considered in this section, means that the material parameters of the *l*-th and $(l+1)$-th layer satisfy

43 $${\frac{{\varepsilon }_{t}}{{\mu }_{t}}|}_{{\omega }_{0},l}\approx {\frac{{\varepsilon }_{t}}{{\mu }_{t}}|}_{{\omega }_{0},l+1}.$$

Under this assumption, the EMFSVA created by the source (42) in the 0-th layer can be obtained from Eqs. (17) and (38) and expressed in the following form, after evaluating all involved integrals:

44 $${{\bf{F}}}_{m}(x,\pm \,|z-z^{\prime} |,t;{\bar{\bar{\varepsilon }}}^{0},{\bar{\bar{\mu }}}^{0},{{\bf{J}}}_{0})=\sum _{\gamma =p,s}\,\frac{{\sigma }_{\gamma }\,{e}^{i{\kappa }_{a}|z-z^{\prime} |-\frac{{x}^{2}}{2{\tilde{\sigma }}_{\gamma }^{2}}}}{{\tilde{\sigma }}_{\gamma }}\,\mathop{\sum }\limits_{n=-\infty }^{\infty }\,{\overline{\bar{{\bf{h}}}}}_{\gamma \pm }({\tau }_{n})\cdot {{\bf{J}}}_{0}(0,{t}_{n}),$$

where $${\tau }_{n}=\frac{\Delta \omega }{2}(t-{t}_{n}-\frac{|z-z^{\prime} |}{{V}_{g}^{a}})$$, $${\tilde{\sigma }}_{p[s]}=\sqrt{{\sigma }_{p[s]}^{2}+\frac{i{a}_{t}^{e[m]}|z-z^{\prime} |}{{a}_{z}^{e[m]}{\kappa }_{a}}}$$, and

45 $${\overline{\bar{{\bf{h}}}}}_{s\pm }(\tau )=[\begin{array}{cc}-\frac{{a}_{t}^{m}\hat{{\bf{y}}}\hat{{\bf{y}}}}{2{\kappa }_{a}} & \pm \frac{\hat{{\bf{y}}}\hat{{\bf{x}}}}{2}\\ \pm \frac{\hat{{\bf{x}}}\hat{{\bf{y}}}}{2} & -\frac{{a}_{t}^{e}\hat{{\bf{x}}}\hat{{\bf{x}}}}{2{\kappa }_{a}}\end{array}]\,{j}_{0}(\tau )-\frac{i\Delta \omega }{2}(\frac{{b}_{t}^{m}}{{a}_{t}^{m}}-\frac{{b}_{t}^{e}}{{a}_{t}^{e}})[\begin{array}{cc}-\frac{{a}_{t}^{m}\hat{{\bf{y}}}\hat{{\bf{y}}}}{4{\kappa }_{a}} & \overline{\bar{0}}\\ \overline{\bar{0}} & \frac{{a}_{t}^{e}\hat{{\bf{x}}}\hat{{\bf{x}}}}{4{\kappa }_{a}}\end{array}]\,{j}_{1}(\tau ),$$

46 $${\overline{\bar{{\bf{h}}}}}_{p\pm }(\tau )=[\begin{array}{cc}-\frac{{a}_{t}^{m}\hat{{\bf{x}}}\hat{{\bf{x}}}}{2{\kappa }_{a}} & \mp \frac{\hat{{\bf{x}}}\hat{{\bf{y}}}}{2}\\ \mp \frac{\hat{{\bf{y}}}\hat{{\bf{x}}}}{2} & -\frac{{a}_{t}^{e}\hat{{\bf{y}}}\hat{{\bf{y}}}}{2{\kappa }_{a}}\end{array}]\,{j}_{0}(\tau )-\frac{i\Delta \omega }{2}(\frac{{b}_{t}^{m}}{{a}_{t}^{m}}-\frac{{b}_{t}^{e}}{{a}_{t}^{e}})[\begin{array}{cc}-\frac{{a}_{t}^{m}\hat{{\bf{x}}}\hat{{\bf{x}}}}{4{\kappa }_{a}} & \overline{\bar{0}}\\ \overline{\bar{0}} & \frac{{a}_{t}^{e}\hat{{\bf{y}}}\hat{{\bf{y}}}}{4{\kappa }_{a}}\end{array}]\,{j}_{1}(\tau ),$$

where the parameters ${a}_{t}^{e[m]}$, ${b}_{t}^{e[m]}$, etc. are expressed through the components of the material dyadics of the 0-th layer, ${\bar{\bar{\varepsilon }}}^{0}$ and ${\bar{\bar{\mu }}}^{0}$.

In order to find the fields in the next layer located at $z\in ({z}_{1},{z}_{2})$ (the 1st layer), we introduce an equivalent Huygens source (a pair of electric and magnetic surface currents) placed at $z={z}_{1}$ (where ${z}_{1} > z^{\prime}$) defined as

47 $${{\bf{J}}}_{m}(x,z,t)={{\bf{J}}}_{1}(x,t)\delta (z-{z}_{1}),$$

48 $${{\bf{J}}}_{1}(x,t)=[\begin{array}{cc}\overline{\bar{0}} & \hat{{\bf{z}}}\times {\bar{\bar{I}}}_{t}\\ -\hat{{\bf{z}}}\times {\bar{\bar{I}}}_{t} & \overline{\bar{0}}\end{array}]\cdot {{\bf{F}}}_{m}(x,{z}_{1}-z^{\prime} ,t;{\bar{\bar{\varepsilon }}}^{0},{\bar{\bar{\mu }}}^{0},{{\bf{J}}}_{0}).$$

The field in the layer $z\in ({z}_{1},{z}_{2})$ can be found from Eq. (44) as ${{\bf{F}}}_{m}(x,z-{z}_{1},t;{\bar{\bar{\varepsilon }}}^{1},{\bar{\bar{\mu }}}^{1},{{\bf{J}}}_{1})$, where ${\bar{\bar{\varepsilon }}}^{1}$ and ${\bar{\bar{\mu }}}^{1}$ are the material dyadics of the 1st layer, from which the equivalent Huygens source ${{\bf{J}}}_{2}$ at $z={z}_{2}$ is expressed by Eq. (48) through ${{\bf{F}}}_{m}(x,{z}_{2}-{z}_{1},t;{\bar{\bar{\varepsilon }}}^{1},{\bar{\bar{\mu }}}^{1},{{\bf{J}}}_{1})$, etc. This procedure is repeated as many times as there are layers at $z > z^{\prime}$, after which the fields in the layers located at $z < z^{\prime}$ can be found in a completely analogous way.

### Numerical examples

#### Negative refraction and focusing by uniaxial MM with hyperbolic dispersion

When the transverse and longitudinal components of the permittivity dyadic of a MM have opposite signs, the isofrequency curves for the $p$-polarized waves are hyperbolas. It is known that the $p$-polarized light refracts negatively when impinging at the interface of a conventional material and such a hyperbolic MM^53,54^. In the following numerical example, we study the implications of this phenomenon on the paraxial propagation of the Gaussian envelopes considered earlier. We shall confirm that the EDGF formalism correctly predicts focusing of a diverging $p$-polarized Gaussian beam by the hyperbolic MM.

At near-infrared frequencies, a hyperbolic MM can be realized, e.g. by embedding vertically aligned metallic nanowires into an isotropic dielectric host. For the sake of a numerical example, here we consider a MM formed by golden nanowires embedded into alumina substrate. By using the Maxwell-Garnett effective medium theory (EMT) for a uniaxial MM formed by such nanowires, the following expressions for the effective transverse and axial permittivities can be obtained^55^:

49 $${\varepsilon }_{{\rm{eff}},t}={\varepsilon }_{h}\frac{{\varepsilon }_{m}(1+f)+{\varepsilon }_{h}(1-f)}{{\varepsilon }_{m}(1-f)+{\varepsilon }_{h}(1+f)},\,{\varepsilon }_{{\rm{eff}},z}={\varepsilon }_{m}f+{\varepsilon }_{h}(1-f),$$

where ${\varepsilon }_{h}$ and ${\varepsilon }_{m}$ are the dielectric permittivities of the host material (Al_2_O_3_^56^) and the plasmonic metal (Au), respectively, and *f* is the nanowires volume fraction. The relative permittivity of gold at near-infrared frequencies follows the Drude dispersion model^57^

50 $${\varepsilon }_{m}(\omega )=1-\frac{{\omega }_{p}^{2}}{\omega (\omega +i{\tau }_{D}^{-1})},$$

where $\hslash {\omega }_{p}=8.5$ eV and ${\tau }_{D}=1.4\times {10}^{-14}$ s. At the frequencies below the plasma frequency ${\omega }_{p}$, ${\rm{Re}}({\varepsilon }_{m}(\omega )) < 0$, and one can achieve ${\rm{Re}}({\varepsilon }_{{\rm{eff}},z}(\omega )) < 0$ with a proper choice of the nanowires volume fraction $f$.

**Figure 1:**
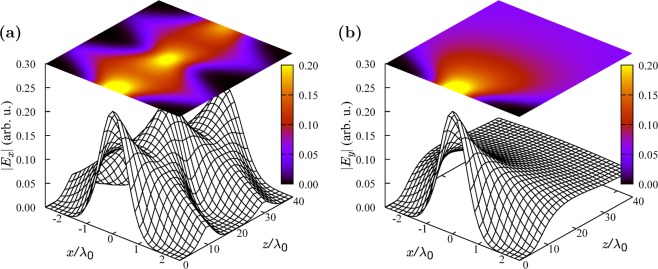
Paraxial propagation of the EMFSVA produced by a source with the Gaussian amplitude profile [Eq. (41)] through a layer of hyperbolic MM sandwiched between two isotropic dielectric layers. (**a**) Amplitude profile of the $p$-polarized beam. (**b**) Same for the $s$-polarized beam.

Let us consider a structure comprised of a hyperbolic MM sandwiched between two isotropic dielectrics with relative permittivity ${\varepsilon }_{d}=3.8$ (e.g. aluminum nitride^56^). The volume fraction of Au nanowires embedded into Al_2_O_3_ substrate is $f=0.15$. The carrier frequency is set to $\hslash {\omega }_{0}=1.32$ eV. An oscillating electric current source with this frequency and the amplitude profile given by Eq. (41) with ${\sigma }_{p,s}={\lambda }_{0}/\sqrt{{\varepsilon }_{d}}$ (where ${\lambda }_{0}=2\pi c/{\omega }_{0}$) and ${\sigma }_{t}\to \infty$ is placed inside the first dielectric at $z=0$. The hyperbolic MM layer is located at $10 < z/{\lambda }_{0} < 30$. At the frequency ${\omega }_{0}$, the relative transverse permittivity of this layer is ${\varepsilon }_{{\rm{eff}},t}=3.8+i6.5\times {10}^{-3}$ and the relative axial permittivity is ${\varepsilon }_{{\rm{eff}},z}=-\,3.8+i0.22$ [Eq. (49)]. The results of the paraxial EDGF-based calculations for this structure for the $p$- and $s$-polarized source currents are displayed in Fig. 1. As one can see, the initially diverging $p$-polarized Gaussian beam, after refracting negatively at the interface $z/{\lambda }_{0}=10$, is focused at $z/{\lambda }_{0}=20$, the middle point of the hyperbolic MM layer, and then it diverges again. After reaching the second interface at $z/{\lambda }_{0}=30$, the beam undergoes another negative refraction and is focused inside the second dielectric layer at the point $z/{\lambda }_{0}=40$. On the contrary, the $s$-polarized beam does not experience any refraction at the MM interfaces and simply diverges. Note that the scales on the $x$-axis and the $z$-axis in Fig. 1 are different, so that the field profile along $z$ is compressed in comparison with that along $x$.

In order to explain how our EDGF formalism is able to reproduce these phenomena in the paraxial approximation, let us consider the expression for the square of the effective beam width, ${\tilde{\sigma }}_{p[s]}^{2}$, at a point $z=z^{\prime\prime}$ inside the MM layer

51 $${\tilde{\sigma }}_{p[s]}^{2}{|}_{z={z}^{^{\prime\prime} }}={\tilde{\sigma }}_{p[s]}^{2}{|}_{z={z}_{1}}+i\frac{{a}_{t}^{e[m]}|z^{\prime\prime} -{z}_{1}|}{{a}_{z}^{e[m]}{\kappa }_{a}}={\sigma }_{p[s]}^{2}+i(\frac{|{z}_{1}|}{{\kappa }_{a,d}}+\frac{{a}_{t}^{e[m]}|z^{\prime\prime} -{z}_{1}|}{{a}_{z}^{e[m]}{\kappa }_{a}}),$$

as follows from Eq. (44). Here, ${z}_{1}=10{\lambda }_{0}$ is the coordinate of the first MM interface and ${\kappa }_{a,d}=2\pi \sqrt{{\varepsilon }_{d}}/{\lambda }_{0}\approx {\kappa }_{a}$ is the propagation factor in the dielectric layer. In the considered hyperbolic MM, ${a}_{t}^{e}/{a}_{z}^{e}\approx -\,1$, while ${a}_{t}^{m}/{a}_{z}^{m}=+\,1$. Therefore, when $|z^{\prime\prime} -{z}_{1}|=|{z}_{1}|$, for the $p$-polarization, the propagation in the dielectric is compensated by the propagation in the MM and thus ${\tilde{\sigma }}_{p}^{2}{|}_{z={z}^{^{\prime\prime} }}\approx {\sigma }_{p}^{2}$, i.e., the $p$-polarized Gaussian beam is refocused at the middle of the MM layer.

#### Negative group velocity and superluminality in active media

In this example, we apply our EDGF formalism to the EMFSVA propagation through a layered structure which is formed by an active medium sandwiched between two passive media. The active layer is the ^132^Xe gas with inverted population, and the passive media are air. The EMFSVA is created by an $s$-polarized surface electric current source located at $z=0$ with the amplitude profile defined by Eq. (41) in which ${\sigma }_{s}\to \infty$ (i.e., only ${k}_{t}=0$ component is present). This source creates the EMFSVA propagating through the three media in the $z > 0$ direction. Because the relative permittivities and permeabilities of the layers are rather close to unity, the layers are well impedance matched and we can apply the theory developed earlier.

The relative permittivity of the ^132^Xe gas with inverted population follows the Lorentzian dispersion with negative oscillator strength^34^:

52 $${\varepsilon }_{t}^{L}={\varepsilon }_{z}^{L}=1-\frac{\eta {\omega }_{p}^{2}}{{\omega }_{r}^{2}-{\omega }_{0}^{2}-i\gamma {\omega }_{0}},$$

where the parameter ${\omega }_{p}/2\pi =0.42$ GHz accounts for both the magnitude of the oscillator strength and the atomic plasma frequency, $\eta =0.9$ is the relative inversion, and ${\omega }_{r}/2\pi =84$ THz and $\gamma /2\pi =4.2$ MHz are the resonant frequency and the linewidth, respectively. We stay detuned from the resonant frequency and set ${\omega }_{0}={\omega }_{r}+{\omega }_{p}/3$. At this point, the group velocity in the active layer is ${V}_{g}^{s}\approx -\,0.97c$ and the assumptions of the EDGF approach hold (see Methods for details).

**Figure 2:**
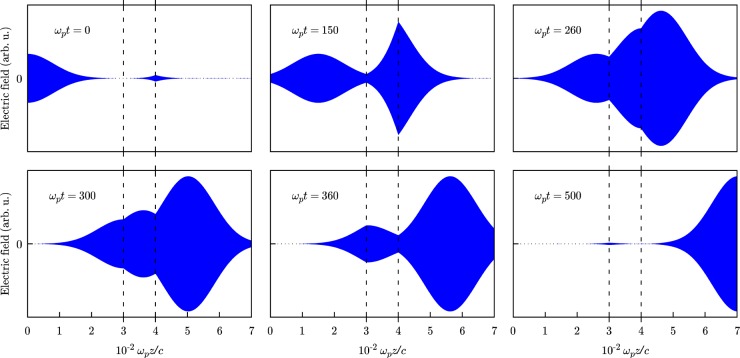
Superluminal propagation of the EMFSVA envelope with normalized duration ${\omega }_{p}{\sigma }_{t}=80$ through a three-layer medium comprised of two air layers at ${\omega }_{p}z/c < 300$ and ${\omega }_{p}z/c > 400$, and an inverted population ^132^Xe gas layer placed in between, plotted at various normalized times ${\omega }_{p}t$ versus normalized axial coordinate ${\omega }_{p}z/c$.

In Fig. 2, we depict the propagation of the EMFSVA envelope with ${\omega }_{p}{\sigma }_{t}=80$ through the three layers (separated by dashed vertical lines in the figure) at various moments of the normalized time: ${\omega }_{p}t=0$, 150, 260, 300, 360, 500. The observed behavior agrees with the one known from literature^34^. As is seen from the top plots in Fig. 2, at ${\omega }_{p}t=0$, the front edge (the precursor) of the Gaussian envelope penetrates into the second layer (the active layer). At ${\omega }_{p}t=150$, the maximum of the original pulse passes forward and the field acquires a noticeable value at the interface of the first and the second layers and, at the same time, we can see that the amplified field in the active layer forms a sharp peak and penetrates into the third layer. We can see how the back propagating pulse is formed in the gain medium and how it interacts with the primary pulse which propagates forward in the first layer at the same interface at ${\omega }_{p}t=260,300,$ and $360$. Finally, at ${\omega }_{p}t=500$, we can see that the main pulse has left the two layers, however, a small effect of its back edge is still present at the interface of the first and the second layers. It should be noted that at ${\omega }_{p}t=260$ the maximum of the pulse exits the second layer earlier than it would do if it had traveled through an equal distance of air, i.e. it appears superluminal. This happens due to the action of the gain medium on the electromagnetic field with a Gaussian-shaped envelope which lacks a definite turn-on moment.

## Discussion

In this work, we have presented the EDGF concept and the related formalism which are applicable for studying the propagation of the amplitude fluctuations of quasi-monochromatic electromagnetic field in anisotropic dispersive media. The developed formalism is aimed to be used in future works to model the dynamics of RHT in such media, in particular, in uniaxial MMs^22^, however, it is equally applicable to the analysis of narrow-band signal propagation in these MMs. Although not considered in this article, the present formulation allows also for a direct finite-difference time-domain-based numerical solution of the dynamic equations for the EMFSVA, which can be useful, e.g. when studying propagation of wave packets in layered MMs.

Note that, in contrast to the conventional dyadic Green’s functions (DGF), our method incorporates the SVA approximations into the linear operator and the source of the Green’s equation. Thus, the EDGF is a wavelet-type dyadic function that describes excitation and propagation of the band-limited *amplitude and phase fluctuations* of the quasi-monochromatic electromagnetic fields. These fluctuations propagate with a velocity that has the meaning of the group velocity. This makes the EDGF approach distinct from the DGF formulations that deal with delta-functional sources in both space and time and result in signal fronts propagating with the speed of light in vacuum, $c=1/\sqrt{{\varepsilon }_{0}{\mu }_{0}}$, due to the fact that in any physical material $\bar{\bar{\varepsilon }}(\omega )\to {\varepsilon }_{0}$, $\bar{\bar{\mu }}(\omega )\to {\mu }_{0}$, when $\omega \to \infty$. Moreover, it can be shown that the EDGF approach inherits from the SVA approach an ability to deal with materials with slow and weak nonlinearity, in which the constitutive parameters depend on the amount of electromagnetic energy passed through the medium^58^.

There is a significant body of literature on the standard DGF in anisotropic and bianisotropic media^59–65^. While in the most general case of bianisotropic medium there is no closed-form representation for the DGF, in the case of uniaxial magnetodielectrics, the time-harmonic DGF can be expressed through a pair of scalar electric and magnetic Green’s functions. When interested in the EMFSVA in such media, a possible approach could be to start from such closed-form representations and expand the frequency-dependent parameters (such as the material dyadics, the propagation factors, etc.) in these relations into series around the carrier frequency ${\omega }_{0}$, with $\Omega =\omega -{\omega }_{0}$ being the small parameter.

Although this would constitute a tractable approach for some cases of uniaxial and bianisotropic media, in this article we have developed a conceptually different method, which can be later extended to media with more general material relations, including nonlinear media (e.g.by using Green-Volterra series^66^). By incorporating the SVA approximations into the material relations and the Maxwell equations, we have formulated the 6-dyadic equation for the EDGF in anisotropic dispersive media and obtained the matrix elements of the EDGF for the uniaxial magnetodielectric media in the Fourier and the configuration spaces. In the case of paraxial propagation, the EDGF for uniaxial media can be written in a closed form, resulting in a formulation similar to the Gaussian beam-based paraxial approximation in optics.

In order to test the ranges of applicability of our formalism, we have considered the propagation of the EMFSVA through a hyperbolic MM and an active medium, sandwiched between two passive dielectric layers. In the former case, the paraxial propagation of the EMFSVA leads to the well-known negative refraction effect^53,54^, while in the latter case one can observe the peculiar phenomenon of negative group velocity associated with superluminal propagation^34^. We have modeled the propagation of the EMFSVA through such media by employing the effective Huygens sources at the interfaces of the neighboring layers. It is worth to mention that the paraxial propagation of the EMFSVA can be used to study the near-field thermal radiation effects in extremely anisotropic media, e.g. in arrays of aligned carbon nanotubes^67^.

We have demonstrated with numerical examples that the developed formalism correctly models negative refraction and focusing by hyperbolic MMs and is also applicable to exotic effects in optical media with inverted population, such as the negative group velocity and superluminality. The group velocity obtained in our formalism agrees with the standard definition known from the literature and results in similar behaviors. The considered examples confirm the applicability and validity of the EDGF approach developed in this paper.

## Methods

### Application of Kotelnikov’s theorem

In its standard formulation, Kotelnikov’s theorem expresses the signal $s(t)$ with a limited spectrum, $\Omega \in [-\frac{\Delta \omega }{2},\frac{\Delta \omega }{2}]$, through a set of the discrete samples, $s({t}_{n})$:

53 $$s(t)=\mathop{\sum }\limits_{n=-\infty }^{+\infty }\,s({t}_{n}){j}_{0}(\frac{\Delta \omega }{2}(t-{t}_{n})),\,{t}_{n}=\frac{2\pi n}{\Delta \omega },$$

where ${j}_{0}(x)=\,\sin (x)/x$. Using this theorem, the convolution integral of two functions, $a(t)$ and $s(t)$, both satisfying Kotelnikov’s spectral condition, can be written as follows

54 $$u(t)={\int }_{-\infty }^{+\infty }\,a(t-t^{\prime} )s(t^{\prime} )\,dt^{\prime} =\frac{2\pi }{\Delta \omega }\,\mathop{\sum }\limits_{n=-\infty }^{+\infty }\,a(t-{t}_{n})s({t}_{n}).$$

We have used this result when obtaining Eq. (17).

### Elements of $\overline{\bar{{\bf{C}}}}$ and $\overline{\bar{{\bf{D}}}}$ matrices

Regarding Eq. (29), the different blocks of ${\overline{\bar{{\bf{C}}}}}_{\gamma ,\pm 1}$ and ${\overline{\bar{{\bf{D}}}}}_{\gamma ,\pm 1}$ ($\gamma =p,s$) matrices are given as follows

55 $${\overline{\bar{{\bf{C}}}}}_{\gamma ,{\rm{sgn}}(Z)}=[\begin{array}{ll}{\bar{\bar{C}}}^{tt,\gamma } & {\bar{\bar{C}}}^{tz,\gamma }\\ {\bar{\bar{C}}}^{zt,\gamma } & {\bar{\bar{C}}}^{zz,\gamma }\end{array}],\,{\overline{\bar{{\bf{D}}}}}_{\gamma ,{\rm{sgn}}(Z)}=[\begin{array}{ll}{\bar{\bar{D}}}^{tt,\gamma } & {\bar{\bar{D}}}^{tz,\gamma }\\ {\bar{\bar{D}}}^{zt,\gamma } & {\bar{\bar{D}}}^{zz,\gamma }\end{array}].$$

Using Eq. (24), recalling ${\kappa }_{0}^{\gamma }$ and ${V}_{g}^{\gamma }$ from Eqs. (31) and (32), respectively, we obtain the non-zero components of $\bar{\bar{C}}$’s and $\bar{\bar{D}}$’s as follows [the structure of these sub-blocks is the same as defined by Eqs. (21–23)]:

56 $${C}_{ee[mm]}^{tt,p[s]}=-\,\frac{{a}_{t}^{m[e]}-{k}_{t}^{2}/{a}_{z}^{e[m]}}{2{\kappa }_{0}^{p[s]}},$$

57 $${C}_{mm[ee]}^{tt,p[s]}=-\,\frac{{a}_{t}^{e[m]}}{2{\kappa }_{0}^{p[s]}},\,{C}_{em}^{tt,s[p]}=[\,-\,]\frac{{\rm{sgn}}\,(Z)}{2},$$

58 $${C}_{ee[mm]}^{zz,p[s]}=-\,\frac{{a}_{t}^{e[m]}{k}_{t}^{2}}{2{a}_{z}^{e{[m]}^{2}}{\kappa }_{0}^{p[s]}},$$

59 $${C}_{ee[mm]}^{tz,p[s]}=\frac{{\rm{sgn}}(Z){k}_{t}}{2{a}_{z}^{e[m]}},\,{C}_{me[em]}^{tz,p[s]}=[\,-\,]\frac{{a}_{t}^{e[m]}{k}_{t}}{2{a}_{z}^{e[m]}{\kappa }_{0}^{p[s]}},$$

60 $${D}_{ee[mm]}^{tt,p[s]}=-\,\frac{{b}_{t}^{m[e]}+{k}_{t}^{2}{b}_{z}^{e[m]}/{a}_{z}^{e{[m]}^{2}}}{2{\kappa }_{0}^{p[s]}}+{V}_{g}^{p{[s]}^{-1}}\frac{{a}_{t}^{m[e]}-{k}_{t}^{2}/{a}_{z}^{e[m]}}{2{\kappa }_{0}^{p{[s]}^{2}}},$$

61 $${D}_{mm[ee]}^{tt,p[s]}=-\,\frac{{b}_{t}^{e[m]}}{2{\kappa }_{0}^{p[s]}}+{V}_{g}^{p{[s]}^{-1}}\frac{{a}_{t}^{e[m]}}{2{\kappa }_{0}^{p{[s]}^{2}}},\,{D}_{em}^{tt,s[p]}=0,$$

62 $${D}_{ee[mm]}^{zz,p[s]}=\frac{{k}_{t}^{2}{a}_{t}^{e[m]}}{2{\kappa }_{0}^{p[s]}{a}_{z}^{e{[m]}^{2}}}(2\frac{{b}_{z}^{e[m]}}{{a}_{z}^{e[m]}}-\frac{{b}_{t}^{e[m]}}{{a}_{t}^{e[m]}}+\frac{{V}_{g}^{p{[s]}^{-1}}}{{\kappa }_{0}^{p[s]}}),$$

63 $${D}_{ee[mm]}^{tz,p[s]}=-\,\frac{{\rm{sgn}}(Z){b}_{z}^{e[m]}{k}_{t}}{2{a}_{z}^{e{[m]}^{2}}},$$

64 $${D}_{me[em]}^{tz,p[s]}=[\,-\,]\frac{{k}_{t}{a}_{t}^{e[m]}}{2{\kappa }_{0}^{p[s]}{a}_{z}^{e[m]}}(\frac{{b}_{t}^{e[m]}}{{a}_{t}^{e[m]}}-\frac{{b}_{z}^{e[m]}}{{a}_{z}^{e[m]}}-\frac{{V}_{g}^{p{[s]}^{-1}}}{{\kappa }_{0}^{p[s]}}).$$

### Smallness parameter

In order to check if it is reasonable to ignore the $O({\Omega }^{2})$ term in Eq. (30), we consider Eq. (25) with an extra second-order term:

65 $${d}_{l}^{e[m]}={a}_{l}^{e[m]}+{b}_{l}^{e[m]}\Omega +\frac{{c}_{l}^{e[m]}{\Omega }^{2}}{2},$$

where $l=t$ or $z$, and substitute it into Eq. (26). We define the smallness parameter ${\delta }_{p[s]}$ as the ratio of the second-order $O({\Omega }^{2})$ term [which is dropped in Eq. (30)] to the first-order $O(\Omega )$ term in the Taylor expansion of Eq. (26) as follows

66 $${\delta }_{p[s]}=\frac{\Omega {V}_{g}^{p[s]}}{2}{\frac{{\partial }^{2}{\kappa }_{p[s]}}{\partial {\Omega }^{2}}|}_{\Omega =0},$$

where in the following calculations we replace $\Omega$ with its maximum value $\Omega =\Delta \omega /2$. Obviously, ${\delta }_{p[s]}$ depends on ${\omega }_{0}$ and $\Delta \omega$, in addition to the material properties. Considering ${k}_{t}=0$, when both polarizations are equivalent, we obtain

67 $${\delta }_{a}=\frac{\Delta \omega }{4{\kappa }_{a}}[{V}_{g}^{a}(\frac{{a}_{t}^{e}{c}_{t}^{m}+{a}_{t}^{m}{c}_{t}^{e}}{2}+{b}_{t}^{e}{b}_{t}^{m})-\frac{1}{{V}_{g}^{a}}].$$

**Figure 3:**
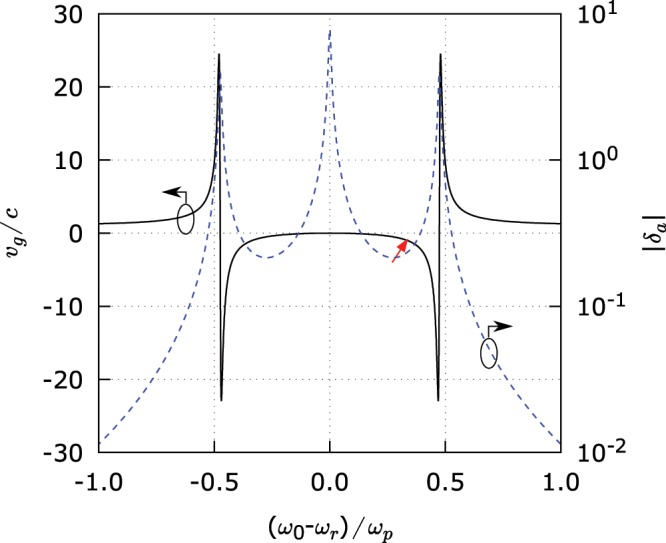
The normalized group velocity ${\upsilon }_{g}/c$ (black solid curve) and the smallness parameter magnitude $|{\delta }_{a}|$ (blue dashed curve) as functions of the normalized frequency shift $({\omega }_{0}-{\omega }_{r})/{\omega }_{p}$. The red arrow indicates the operating point corresponding to the pulse propagation shown in Fig. 2.

In Fig. 3, we depict the normalized group velocity and the smallness parameter versus the normalized frequency shift (for the case of the superluminal propagation considered in Numerical examples). In this figure, ${\upsilon }_{g}={\rm{Re}}({V}_{g}^{a})$ and ${\delta }_{a}$ are measured by the scales on the left and the right, respectively. As can be seen from Fig. 3, $|{\delta }_{a}|\approx 0.2$ near the operational frequency ${\omega }_{0}={\omega }_{r}+{\omega }_{p}/3$ for the considered example. This value can be considered sufficiently small. On the other hand, in the regions where the group velocity becomes extremely superluminal, we can see that $|{\delta }_{a}|\gg 1$, which indicates that in these regions the pulse propagation is very much affected by the group dispersion and the group velocity completely looses its physical meaning, even if understood in a purely kinematic sense.
